# Adopting Individualized Strategies to Prevent Large-For-Size Syndrome in Adult Liver Transplant Recipients: The Graft Morphology Should Also Be Taken Into Account

**DOI:** 10.3389/ti.2022.10683

**Published:** 2022-09-05

**Authors:** Guang-Peng Zhou, Lin Wei, Zhi-Jun Zhu

**Affiliations:** ^1^ Liver Transplantation Center, National Clinical Research Center for Digestive Diseases, Beijing Friendship Hospital, Capital Medical University, Beijing, China; ^2^ Clinical Center for Pediatric Liver Transplantation, Capital Medical University, Beijing, China

**Keywords:** large-for-size syndrome, *ex vivo* right posterior sectionectomy, size mismatch, graft morphology, right anteroposterior vertical distance

Large-for-size syndrome (LFSS) is a less common but life-threatening complication following adult liver transplantation during the early post-transplant period, characterized by postoperative liver necrosis, vascular complications, and primary nonfunction due to severe liver graft compression (1). In this issue of Transplant International, Pu et al. reported a novel surgical technique of *ex vivo* right posterior sectionectomy while preserving the right hepatic vein in the liver graft to prevent posttransplant LFSS in adult liver transplant recipients, which was successfully performed in all five recipients discharged without procedure-related complications (2). Pu et al. should be congratulated for describing a feasible intervention to save patients from the potential risk of LFSS. However, the graft morphology should also be taken into account when adopting this novel surgical treatment.

Pu et al. selected graft-recipient weight ratio (GRWR) combined with graft weight (GW)/right anteroposterior (RAP) as a new “LFSS predictor,” in which both GRWR > 2.5% and GW/RAP > 100 g/cm indicated the need for reduction of the right liver graft. However, this new “LFSS predictor” has its intrinsic limitation, which only considers the graft weight and the depth of the lower right hemithorax of the recipient, but not the morphological parameters of the graft, especially the RAP vertical distance and the longest horizontal distance (3). The morphology of the liver grafts may differ among individuals. Some large-volume livers exhibit a short, “squat” shape (relatively short and thick right liver span). In contrast, others have a narrow, flat, and elongated morphology (relatively long and thin right liver span) (3). Therefore, both GRWR and GW/RAP could not fully indicate the possibility of severe compression of the right liver graft from the recipient’s rib cage. Within the past several years, our center has also completed several *ex vivo* right posterior sectionectomy cases in both pediatric and adult liver transplant recipients. Despite the advantages of *ex vivo* right posterior sectionectomy, as described by Pu et al., it must be admitted that this surgical procedure still carries increased risks of surgical complications, especially in patients with decompensated cirrhosis with a high MELD score (1, 4). Based on our experience and others, the size discrepancy between the anteroposterior dimensions of the graft and the longest RAP of the recipient should still be considered a first-line index for evaluating the occurrence of the LFSS. Thus, the choice of the liver segments to be resected should be based on the combination of the anthropometrics of the donor graft with that of the recipient (1, 5). The anteroposterior dimension of the graft can be accurately measured on the back table to provide a precise parameter for determining the necessity of right posterior sectionectomy. If the right liver graft vertical distance is less than the longest RAP vertical distance of the recipient, graft reduction with resection of the right posterior sector (segment 6–7) may not be necessary. In this condition, left lateral lobectomy or left hemihepatectomy may be more appropriate with their convenience and relatively low risk (6). Nevertheless, concerns remain that a limited graft reduction such as left lobectomy is very unlikely to avoid rib compression over the right liver (3). Recently, Paterno et al. provided a further solution named “bilateral marginal costotomy,” which rescued a liver transplant recipient from severe graft compression from the bilateral narrow rib cages after the failed temporary abdominal closure (7). Thus, marginal costotomy can be performed either as a primary or adjunctive treatment to avoid graft compression due to the ribs after the implantation of a large-for-size liver graft or as a rescue treatment after conventional interventions failed to relieve allograft compression.

Collectively, combining the volumetric and morphological parameters of the donor liver with the anthropometrics of the recipient may be more beneficial in determining the individualized strategies to prevent the occurrence of LFSS in adult liver transplant recipients when facing donor-recipient mismatching.

## Data Availability

The original contributions presented in the study are included in the article/supplementary material, further inquiries can be directed to the corresponding author.
